# Burden of motorcycle-related injury in Malaysia

**DOI:** 10.1186/s12245-015-0065-4

**Published:** 2015-06-04

**Authors:** Nik Hisamuddin NA Rahman, Kamarul A Baharuddin, Syarifah Mastura S Mohamad

**Affiliations:** School of Medical Sciences, USM, Kota Bharu, 16150 Malaysia; School of Health Science, USM, Kota Bharu, 16150 Malaysia

**Keywords:** Injury, Road safety, Motorcycle, Trauma

## Abstract

**Background:**

Road traffic injury (RTI) contributes to major morbidity and mortality in both developed and developing countries. Most of the injuries are caused by road-related injuries that specifically relate to motorcycle crash. We attempted to conduct a short survey to determine the magnitude of burden related to motorcycle-related RTIs in Malaysia. We hypothesize that motorcycle-related RTI in Malaysia contributes significantly to the health burden in the country.

**Methods:**

The cross-sectional survey involves data searching related to RTI in Malaysia from the relevant agencies such as the Ministry of Health Malaysia, Royal Police Force, and Malaysia Institute of Road Safety Research (MIROS) through their official websites and PubMed search. The three agencies are well established and recognized by the Malaysian government in dealing with data collection for the injury nationwide. The primary aim is to determine the prevalence of motorcycle-related RTI, and secondary outcomes are the overall mortality and the contributing factors.

**Results:**

Of the cause of trauma, 80 % is due to RTI, and the most vulnerable road users such as pedestrians and motorcyclists are affected the most. Of all RTI, 70 % is contributed by the motorcycle crash, and there are a significant number of deaths for both rider and pillion rider of the motorcycle than for other types of vehicles. Human error is the main reason to be blamed, specifically the attitude of the riders on the road.

**Conclusions:**

Trauma is one of the common reasons for death and hospitalization in Malaysia. Motorcycle-related RTI in Malaysia contributes significantly to the health burden in Malaysia. The Malaysian government and non-government agencies have worked together seriously in implementing a preventive measure to reduce the incidence and aftermath of motorcycle-related RTI. However, data is still lacking, and every effort is made to increase the amount of research in the field.

Strengths of the article are as follows:

- Latest alarming data on motorcycle-related injuries in the developing country.
- The data is collected from multi-agencies recognized by ministries in the country.
- Very limited publication specifically on motorcycle-related injuries is available.

Limitations of the article are as follows:

- The data is only from one country.
- The statistical data is gathered from a variety of sources, i.e., relevant agencies and authorities and website of the involved ministries.

## Background

Road traffic injury (RTI) contributes to major morbidity and mortality in both developed and developing countries. Malaysia spans over 330,289 km^2^. Its 29.33 million population in 2012 comprises of 51 % male and 49 % female. The majority are Malays and indigenous Malay ethnic group (66.1 %). The majority of the population is between the age of 15 and 64 years (68.3 %). The annual population growth rate is 1.3 %. This yields a population density of 85 per km^2^. The life expectancies in Malaysia for males and females were 72 and 76 years old, respectively [1]. Malaysia is categorized as a middle-income country, with approximately US$ 13,740 per capita gross domestic product (GDP). However, the government spends only 7.7 % of its annual budget on health care (US$ 6.33 billion) most of which is utilized for the operation of the health system (89 %). The remainder is used for the development projects nationwide [2]. In Malaysia, injury is one of the top six leading causes of hospital admission and deaths after ischemic heart disease, cerebrovascular disease, septicemia, neoplastic disease, and pneumonia (Table 1) [3]. Most of the injuries are caused by road-related injuries specifically related to motorcycle crash. We attempted to conduct a short survey to determine the magnitude of burden related to motorcycle-related RTI in Malaysia.

**Table 1:** MOH statistics on the top 10 causes of hospital admission in Malaysia

Ten principal causes of hospitalization in Malaysian hospitals, 2012		
1.	Pregnancy, childbirth, and the puerperium	27.32 %
2.	Diseases of the respiratory system	11.02 %
3	Injury, poisoning, and certain other consequences of external causes	8.22 %
4.	Diseases of the circulatory system	7.55 %
5.	Certain conditions originating in the perinatal period	7.55 %
6.	Certain infectious and parasitic diseases	6.82 %
7.	Diseases of the digestive system	4.88 %
8.	Diseases of the genitourinary system	4.48 %
9.	Factors influencing health status and contact with health services	3.64 %
10.	Neoplasms	3.34 %

## Methods

The cross-sectional survey involves data searching related to RTI in Malaysia from the relevant agencies such as the Ministry of Health Malaysia, Royal Police Force, and Malaysia Institute of Road Safety Research (MIROS) through their official websites and PubMed search. The primary aim is to determine the prevalence of the motorcycle-related RTI, and secondary outcomes are the overall mortality and the contributing factors. The fatality cases include those who died immediately at the scene and late death in hospitals after sustaining motorcycle-related RTI.

This article does not require ethical approval because it is a brief review report article and does not involve data collection from patients.

## Results

Road traffic accidents (RTA) contribute to most of the injury cases in Malaysia. There were over 400,000 reported cases of RTA according to the Malaysian Police Force statistics year 2011 (Table 2).

**Table 2:** Total casualties and damages caused by RTA in Malaysia, 2002–2011

Year	Total number of accidents	Casualties			Total
		Death	Serious	Minor	
2002	279,237	5,891	8,425	35,236	49,552
2003	298,653	6,286	9,040	37,415	52,741
2004	326,817	6,228	9,229	38,631	54,088
2005	328,264	6,200	9,397	31,429	47,026
2006	339,252	6,287	9,254	19,884	35,425
2007	363,319	6,282	9,273	18,444	33,999
2008	372,990	6,527	8,866	16,901	32,294
2009	397,330	6,745	8,849	15,823	31,417
2010	414,421	6,872	7,781	13,616	28,269
2011	449,040	6,877	6,328	12,365	25,570

Source: Royal Malaysian Police

Statistically, there is an average 19 deaths due to RTA daily, a phenomenon that is very alarming. According to the World Health Organization (WHO) ranking, Malaysia is at the 20th place in the world for death ranking due to RTA. The latest WHO report shows that the death rate in Malaysia due to RTA is 34.5 for every 100,000 population [4]. The majority of the RTI victims involve the most vulnerable group of road users namely pedestrians, motorcyclists, and their pillion riders. Motorcycles are a relatively cheap and reliable mode of transportation in Malaysia. In the year 1990, the number of registered cars and motorcycles was 1,811,141 and 3,035,930, respectively. In 2007, the number yet increases to 7,419,643 for cars and 7,943,364 for motorcycles [5]. This increasing number of vehicles affects the density of vehicles per road and thus the number of mishaps at a particular area (Table 3).

**Table 3:** The total number of vehicles involved in RTA in Malaysia, 2002–2011

Year	Motorcycle	Motorcar	Van	Bus	Lorry	Four-wheel drive	Taxi	Bicycle	Others	Total
2002	86,779	320,649	19,077	9,256	37,794	14,783	6,187	3,278	10,043	507,843
2003	95,545	351,832	20,277	9,673	42,753	16,429	6,632	2,993	9,500	555,634
2004	99,227	388,589	20,086	9,265	45,420	18,306	7,111	2,963	11,186	602,153
2005	97,072	376,061	19,085	8,594	42,062	19,106	7,043	2,751	9,362	581,136
2006	104,107	411,444	20,428	9,700	44,767	20,885	7,751	2,834	12,266	634,182
2007	111,765	426,941	21,109	10,285	47,696	21,823	8,809	2,690	14,909	666,027
2008	111,819	435,665	20,392	9,356	48,250	22,793	8,769	2,463	11,571	671,078
2009	113,962	472,307	19,220	9,380	46,724	23,581	8,669	2,486	9,294	705,623
2010	120,156	511,861	18,788	9,580	50,438	25,777	9,899	2,178	11,756	760,433
2011	129,017	546,702	17,916	9,986	53,078	30,828	11,197	2,033	16,394	817,151

Source: Royal Malaysian Police

Of all RTA victims in 2012, approximately 70 % involved motorcyclists. Motorcyclist-related fatalities accounted for 60 % of all road fatalities. Interesting facts are that most of these victims were young adolescents, and many of them did not hold any valid driving license [6]. In 2008, 428,475 cars and 111,958 motorcycles were reported to be involved in accidents. The total fatalities in the same year were 6287. Of those who died, 58.7 % involved the motorcycle riders and pillions. There is a significant number of deaths combined from both rider and pillion rider of motorcycles than from any other type of vehicles. In terms of admission to hospitals in Malaysia, motorcycle-related injuries are the leading cause of admission (79.9 %) followed by motorcars (10.7 %) and others 9.4 % [7]. This is further strengthened by the Malaysian national trauma database findings which reported that 64.9 % of the trauma is due to motorcycle [8]. A similar trend of motorcyclist contribution to the RTI statistics is observed in other countries in the Southeast Asia (SEA) region (Table 4).

**Table 4:** Deaths by type of road users in various regions 2012

No	ASEAN countries	Population	Registered motorcycle (2012)		Reported fatalities (2012)		Road fatalities per 100,000 population	Motorcycle fatalities per 10,000 registered motorcycles
			Total (million)	(%)	Total	(%)		
1	Brunei	390,056	0.01	4	54	11	13.8	4.9
2	Singapore	4,436,281	0.14	17	214	48	4.8	7.1 (3)
3	Lao P.D.R.	5,859,393	0.51	79	608	80	10.4	9.6 (2)
4	Cambodia	14,443,679	0.13	84	1545	63	10.7	75.1 (1)
5	Malaysia	26,571,879	7.91	47	6282	58	23.6 (1)	4.6
6	Myanmar	48,798,212	0.68	65	1638	10	3.4	2.4
7	Thailand	63,883,662	16.14	63	12,493	70	19.6 (3)	5.4
8	Vietnam	87,375,196	21.78	95	12,800	80	14.6 (3)	4.7
9	Philippines	87,375,196	2.65	48	1185	37	1.3	1.7
10	Indonesia	231,626,978	46.22	73	16,548	61	7.1	2.2
	Total	571,345,453	96.17	58	53,366	52	-	-

## Discussion

Motorcyclists are particularly vulnerable to injury, as they do not have the steel car frame to absorb the transmitted forces imparted during a collision. There is a massive amount energy transferred to the motorcyclists upon impact, and there are three possible mechanisms by which a motorcyclist may be injured [9]. The first would be by frontal impact or ejection that occurs when the part of the motorcycle is abruptly stopped upon impact while the rest of it, together with the rider, continues to move. The axle acts as the center of gravity which tips the motorcycle forward and the rider would go over the handlebars. The impact of the head, thorax, or abdomen onto the handlebars may result in blunt abdominal injuries as well as traumatic rupture of the abdominal wall. The femurs may also be fractured if the rider’s feet remain strapped onto the footrests, which result in direct impact of the femurs onto the handlebars. The second mechanism is lateral impact or ejection, which may result in open or closed fractures of the extremities of the impacted side. As the rider lands, secondary injuries may then follow. The third mechanism is when a motorcyclist attempts to slow the motorcycle down from the impending impact by turning it sideways (90°) and dropping it to the ground. The “down” leg is at risk for significant soft tissue injuries and fractures. A short survey was conducted in 2012 among all the motorcycle-related RTI in two tertiary centers in Kelantan, Malaysia (Dr Aehtoosham Suleman, Type of injury presentation of traumatic motorcyclists at the emergency department in Malaysia, unpublished dissertation 2012). The investigators noted that the most commonly injured body region was the extremity that occurred in 80.0 % of the study population. A large number of subjects sustained lower extremity injury (82 %) as compared to upper extremity injury (65 %), and the commonest injury pattern among these subjects were superficial wounds (65.3 %) followed by fractures (16.3 %). A similar trend is noted with injuries involving the upper extremities. The third most common injury among the motorcyclists was head injury. This ranged from superficial wounds to intracranial injury. The above finding is also observed by Pang et.al in their study on 226 non-fatal motorcyclists in Malaysia. In their study, it was reported that limb injuries outnumbered that of head, thorax, and abdomen and ranked the highest for the indication of hospitalizations among the study population (Table 5) [10]. In another study conducted in Penang, Malaysia, 140 RTA fatality cases were identified in 2007 that underwent postmortem at the forensic department of a general hospital. Of the victims who died before reaching hospital, 57 % were among the motorcyclists. Of those who died, 92 % sustained severe traumatic brain injury. The average AIS score for these victims was 6 (Dr Noor Azleen Ayop, Pattern of injury and preventability of pre hospital death among motorcyclists, unpublished dissertation 2012). However, further data collection nationwide is required to strengthen the finding of this survey. Unfortunately, we found that there is no uniform injury severity recording carried out among all the agencies. The police force subdivided the injury into very generic groupings namely mild, moderate, severe, and death. The only reliable clinical severity scoring is available from hospital data.

**Table 5:** Pattern of injuries sustained by motorcyclists in 2012 in ED of two tertiary centers

AIS body regions	Percentage (%)
Head and neck	31.7 %
Face	13.3 %
Thorax	5.0 %
Abdomen	5.0 %
Extremities	80 %
External	35 %

### Preventive efforts and research

There are many ways to tackle this serious issue, one of which is the implementation of preventive and road traffic safety programs. Malaysia has also experienced significant change in ways to tackle road safety. Few important organizations start to integrate and work on the same issue in promoting road safety. Those include the Malaysian Police Force (PDRM), Ministry of Transportation, Malaysian Institute of Research on Road Safety (MIROS), Department of Road Safety (JKJR), Department of Road and Transportation (JPJ), Ministry of Work, and Ministry of Health [11, 12].

Despite a lot of advancement in the modern engineering of the motorcycle, man has failed to put effective protective equipment and measures to protect the rider in the motorcycle itself. The initiative of any protection is concentrated on the rider himself rather than on the motorcycle. Thus, the rider needs to utilize protective equipments such as helmet, leather protective gears, boots and gloves, and conspicuous clothing. An essential checking of the brake light and wheel pressure besides keeping the front light switch on all the time may ensure further protection for the rider [13]. Legislation in Malaysia mandates the rider to switch on the light at daylight in order to make the rider more visible and reduce fatalities among motorcycle users [14, 15]. In Malaysia, wearing a crash helmet or a safety helmet is compulsory and mandatory by law. The helmet has its own standard for its manufacturing and its safety regulation, and in Malaysia, the bodies that ensure helmets safety is the Standards and Industrial Research Institute of Malaysia (SIRIM) [16].

A preventive measure requires considerable amount of data to elicit the current problem. Data obtained from robust studies can be used to create preventive measures and hence reduces the incidence of motorcycle-related RTI. Unfortunately, due to the limited number of prospective studies conducted in Malaysia and the lack of an integrated sustainable injury registry, neither the accurate morbidity nor the mortality of road-related motorcycle injuries has been recorded nationally. Ambak et al. conducted a cross-sectional study in Selangor, Malaysia, in 2011 to examine the percentage of compliance rate regarding helmet use and to identify its characteristics [17]. The observations among 1150 motorcyclists show that only 46.6 % used helmets properly, 10.6 % untied helmet, and 42.8 % did not wear a helmet. The percentage of improper helmet usage in the locations of study was considered high, and it seems those helmet initiative programs are insufficient to overcome the problem. Therefore, there is a serious need to introduce a new mechanism or method that can be utilized to incorporate behavior adaptation toward safety concern among motorcycle users. In 2006, Law et al. conducted an interesting cross-sectional analysis among motorcyclists who are labelled as red light runners in Kuala Lumpur, Malaysia [18]. Interestingly, they found that most of the red light running violations occurred at the signalized intersections with high-motorcycle volume, shorter change interval time, shorter amber time, and longer cycle length. The findings can be used to evaluate the effectiveness of specific countermeasures, such as providing a longer change interval time and amber time, shorter cycle length, less number of signal phase, or all of them, for different traffic volumes at signalized intersections.

In 2005, a group of researchers from MIROS analyzed all motorcyclists injured who sustained cervical spine injuries in Kuala Lumpur [19]. The types of injuries sustained were acquired from medical reports. Information on the crash scene and crash mode was obtained from police reports, and interview sessions were arranged with the motorcyclists involved in the crash. Generally, a high count was noted for injuries to the lower cervical vertebrae, especially at vertebrae C5, intervertebral C5-C6, and vertebrae C6. The upper cervical spine was observed to have a high frequency of injury at C2, especially the odontoid process. Neck flexion and extension movements are the most frequent neck injury mechanisms, especially in frontal- and rear-end-impacted motorcycles. This has proven that the mechanism of impact during the crash greatly influenced the clinical finding, and every paramedic and doctor involved in treating the victims should be made aware of the biomechanics of the injury so that they do not miss the serious injury.

There are many more ongoing researches in the same field such as the geo-mapping of the RTA, the development of the electronic road traffic injury surveillance system, and the biomechanics of injury. The research effort must involve multi-agencies, and grant support from the government must be made available continuously for the sustainable preventive projects in the future. Another area that requires more attention by the law maker is the development of a sustainable and funded nationwide data collection system such as a trauma registry. At present, unfortunately, this database system is not available nationwide but more of the regional collection which is either a manually or electronically hospital-based system. However, the police force has established a nationwide and centralized data collection on all motor vehicle crashes that are reported to them. The dataset includes the prehospital data, mechanism of crashes, severity of injury (based on body parts injured), and clinical outcome. Effort has been made to integrate data collection between agencies such as hospitals, MIROS, and the police force which has a uniform dataset definition. Even though the data mentioned above came from various sources, these published data represent prevalence among population of Malaysia and can be considered accurate enough since they were extracted from established and recognized agencies such as MIROS, the Ministry of Health, and the Royal Police Force of Malaysia. Unfortunately, we do not have the exact figure on the economic impact of trauma care on the RTI victims. This is probably another aspect that can be looked upon seriously by the government in tackling the burden of motorcycle-related injury holistically.

### Limitations

The survey obtained the data mainly from official websites and previous publication through PubMed. There is a tendency for missing data of the actual incidence of the motorcycle-related RTI and the underreporting of the factors contributing to the crash. The data is only up to the year 2012. However, the data published is from reliable and well-maintained sources and still reflects the present situation in the country.

## Conclusions

Trauma is one of the common reasons for death and hospitalization in Malaysia. Motorcycle-related RTI in Malaysia contributes significantly to the health burden in Malaysia. The Malaysian government and non-government agencies have worked together and seriously in implementing the preventive measure to reduce the incidence and the aftermath of the motorcycle-related RTI. However, data is still lacking, and every suitable effort is taken to increase the amount of research in the field.

### Data sharing

Extra data and information is available by emailing to nhliza@hotmail.com (Attention Dr. Nik H Rahman). Extra data can also be obtained from websites as follows:

- Ministry of Health Malaysia at http://www.moh.gov.my/images/gallery/stats/heal_fact/health_fact_2012_page_by_page.pdf
- Ministry of Transport at http://www.jpj.gov.my/web/eng/statistic
